# Experiences of New Zealand Māori Mothers’ Engagement with Health and Social Services Post-COVID-19 2020 Lockdown

**DOI:** 10.1007/s40615-025-02419-4

**Published:** 2025-04-22

**Authors:** Nikki M. Barrett, Lisette Burrows, Polly Atatoa-Carr, Linda T. Smith

**Affiliations:** 1 Te Ngira: Institute for Population Research, University of Waikato, Hamilton, New Zealand; 2 Te Whare Wānanga O Awanuiārangi, Whakatāne, New Zealand

**Keywords:** Maternity care, Māori wāhine, Indigenous health, Childbirth, Kaupapa Māori, Mana wahine

## Abstract

**Background:**

Despite universal provision of maternity care, Māori (Indigenous peoples of Aotearoa/New Zealand) experience significant maternal and infant health disparities compared to their dominant Pākehā (non-Māori) counterparts. This paper examined the lived realities of postnatal Māori māmā (mothers) engaging with health and social services. Enablers and barriers were identified to better understand what is required to strengthen health services’ responsiveness to Māori māmā health needs and aspirations.

**Methods:**

Underpinned by Kaupapa Māori research principles, which are grounded in Māori cultural values, emphasising self-determination, and Māori aspirations, a small cohort of 17 expectant Māori māmā were recruited from a Māori childbirth education programme to participate in a three-phase study. Phase three, the focus of this paper, involved seven semi-structured, open-ended telephone interviews with Māori māmā. A thematic analysis, underpinned by a mana wahine (authority inherent in Māori women) theoretical perspective amplified these experiences.

**Results:**

Five themes were identified that encapsulated participants’ engagement and interactions with health and social services. These themes were as follows: (1) right to enact tino rangatiratanga (autonomy) and self-achievement; (2) responsiveness of services; (3) service and system issues; (4) need for greater choice and opportunity; and (5) impact of COVID-19.

**Conclusions:**

This study privileged the voices of Māori wāhine, highlighting their experiences with a complex and often unresponsive health system. Participants valued services that enabled them to exercise tino rangatiratanga. Echoing the experiences of other Indigenous Peoples, incorporating culturally relevant practices into perinatal health services is crucial for achieving health equity and addressing disparities.

**Supplementary Information:**

The online version contains supplementary material available at 10.1007/s40615-025-02419-4.

## Background

In Aotearoa/New Zealand (hereafter Aotearoa), free maternity services are provided to all citizens and permanent residents [1]. Despite universal provision of maternity care, Māori, who comprise of 17% of the population, experience significant maternal and infant health disparities compared to their dominant Pākehā counterparts [2]. Māori infants make up 30% of live births but have significantly higher infant mortality rates [3]. Māori infants are overrepresented in other health indicators, such as greater exposure to cigarette smoke and alcohol whilst in utero, lower-birthweight, significantly higher unintentional injury hospitalisation rates, higher rates of hospital admission for respiratory illnesses, and lower childhood immunisation rates, and they are significantly less likely than non-Māori babies to have been exclusively breastfed when they were 3 months (13 weeks) old and 6 months (26 weeks) [4–6]. These stark inequities breach the obligations of the nation’s foundational document, Te Tiriti o Waitangi as well as the Declaration of Indigenous Rights which both advance a need for equitable health outcomes [7–9].

### Māori Maternities and Health System in Aotearoa

The current health system and subsequent maternity services have been predominantly shaped by western ideologies and practices, centred around hospital institutions and medical intervention [10]. This contrasts with traditional maternity practices predating colonisation, where Māori communities had established, and culturally significant, maternity structures. Three major factors contributed to the suppression of Māori knowledge and practices.

First, the introduction of Western policies and legislation that dismantled Māori knowledge systems and processes. The work of Māori healers (Tohunga) was outlawed by the Tohunga Suppression Act 1907. The introduction of the Midwives Registration Act 1904 made it ‘…illegal for anyone not trained under a Western system of midwifery to practise as a midwife, effectively ruling out traditional birth attendants…’ [11] (p. 74). This meant that Tohunga, who were central to labour and birthing, were forcibly replaced with predominately non-Māori midwives.

Second, prohibiting the use of home births, including ancestral home (marae) [12] and enforcing the use of hospitals [13], negatively impacted how Māori women (wāhine) laboured and birthed their baby (pēpi). The forced use of hospitals meant wāhine were relocated from a place of safety and security to an alien environment that was unfamiliar and ignored cultural protocols, principles, and beliefs (tikanga), ultimately disregarding Māori needs and aspirations.

Third, the assimilation of Western family structures redefined the position and function of Māori wāhine [14]. Traditionally, Māori society was organised around extended family groups, or whānau, where wāhine held significant power and responsibility in decision-making, leadership, and cultural practices. With the imposition of Western family structures, which emphasised patriarchal norms and nuclear families, the roles of Māori women were marginalised, reducing their influence and diminishing their value, creating a superior/inferior complex between men and women. The Western ideals of birthing have marginalised the role of wāhine, irradicating the sacredness (tapu) of the maternal body [12].

In Aotearoa, women are supported by a Lead Maternity Carer (LMC), often a midwife, who is clinically responsible for providing primary maternity care during the antenatal period, labour, birth, and up to 6 weeks postnatally [15]. Registered must apply for an Annual Practising Certificate (APC) to practice, with The Midwifery Council of New Zealand also requiring midwives to complete a recertification programme every 3 years [16]. LMC midwifery care is available at no cost to citizens and eligible residents of Aotearoa, regardless of the chosen birth setting. Women can opt for home births, hospital births (including secondary or tertiary hospitals), or midwife-led primary birthing units.

Colonisation affected all aspects of traditional Māori maternities [12] and continues to negatively impact Māori maternal and infant health outcomes [17–19]. Came et al. [20] argue that the colonial policies (generic or mainstream policy) that are designed for ‘all’ people in Aotearoa, privilege non-Māori but have negative implications for Māori health. Aotearoa’s colonial history impacts health services with Māori experiencing all levels of racism.

Institutional racism involves ‘a pattern of differential access to material resources, cultural capital, social legitimation and political power that disadvantages one group, while advantaging another’ [4] (p. 21). Makowharemahihi et al.’s [21] study counters dominant discourse that Māori do not engage with health services and argues that Māori wāhine do engage early; however, system barriers lead to avoidable delays in accessing the prescribed maternity pathway of care.

Interpersonal racism involves prejudice and discrimination based on race, where prejudice refers to different assumptions about people’s abilities and motives, and discrimination refers to different actions towards them [22]. Studies have shown that Māori and other minority ethnic groups experience significant prejudice and discrimination, adversely affecting māmā and pēpi. For example, Bécares and Atatoa-Carr [23] found a strong correlation between ethnically motivated attacks by health professionals on Māori, Pacific, and Asian women and postnatal depression. Similarly, Thayer et al. [24] found that perceived racism led to lower birth weights and shorter gestation periods amongst Māori wāhine compared to non-Māori.

Finally, internalised racism is defined as ‘acceptance by members of the stigmatised races of negative messages about their own abilities and intrinsic worth’ [22] (pp. 1213). Houkamau et al. [25] argue that deficit framing and language discourse of being labelled *at risk* and *vulnerable* impedes Māori engagement with health services, affects how Māori are perceived by health professionals and, importantly, how Māori see themselves. Masters-Awatere and Graham [26] argue that Māori have come to expect low standards of care, suggesting that dominant discourses directly impact possibilities for achieving optimal health.

Lack of access to quality health care services is a contributing factor to Māori maternal inequities. Pihama’s [27] study found young Māori wāhine are often described as being *problematic* and *high risk*, especially as teenage mothers. This view is also supported by Lawton et al. [28] who claims ‘young Māori mothers experience the stigma of being Māori and being teenage mothers. Their babies also experience poorer health outcomes than non- Māori’ (p. 247). Stevenson et al. [29] found that ‘poor communication, separation from baby and/or whānau [family], and poor access to support services impact negatively on birth experiences’ (p. 135).

These events of racism are consistent with findings from other Māori health studies focused on engagement and interactions with health services [30–32] as well as empirical evidence from studies involving other Indigenous Peoples and their experiences with health services [33–36]. Findings from these and other studies emphasise the need for Indigenous, or in the context of Aotearoa, Kaupapa Māori interventions that prioritise end-user aspirations to obtaining optimum health [2, 37].

### Research Study

Kaupapa Māori research (KMR) is centred on revolves issues significant to Māori communities, underpinned by Māori self-determination, sovereignty, and aspirations [38]. Smith’s [39] further work explains that this transformative approach gives precedence to Māori knowledge and ways of being, supporting community aspirations, development, and sovereignty.

Mana wahine, an extension of KMR theory, is an approach that seeks to privilege the voices and stories of Māori wāhine. At its core, mana wahine is about ‘making visible the narratives and experiences, in all of their diversity, of Māori women’ [40] (p. 11). Though Kaupapa Māori and mana wahine academic scholarship is growing, these studies are limited in number, resulting in a lack of Māori voice in academic scholarship. Specifically, there is a shortage of scholarship centred on postnatal experiences of Māori māmā (mothers).

In Aotearoa, best practice models that inform policy are premised on Western evidence-based research. A lack of Māori voice in academic scholarship is concerning particularly when supposed best practice models are adopted using evidenced-based research [41]. Furthermore, the unprecedented global pandemic, COVID-19, that has disrupted delivery of health services, could widen the equity gap between Māori and non-Māori if Māori needs are not prioritised [42]. As such, there is urgency for the voices of Māori māmā in academic scholarship to be privileged to ensure health policy is responsive to Māori needs and aspirations.

**Fig. 1:**
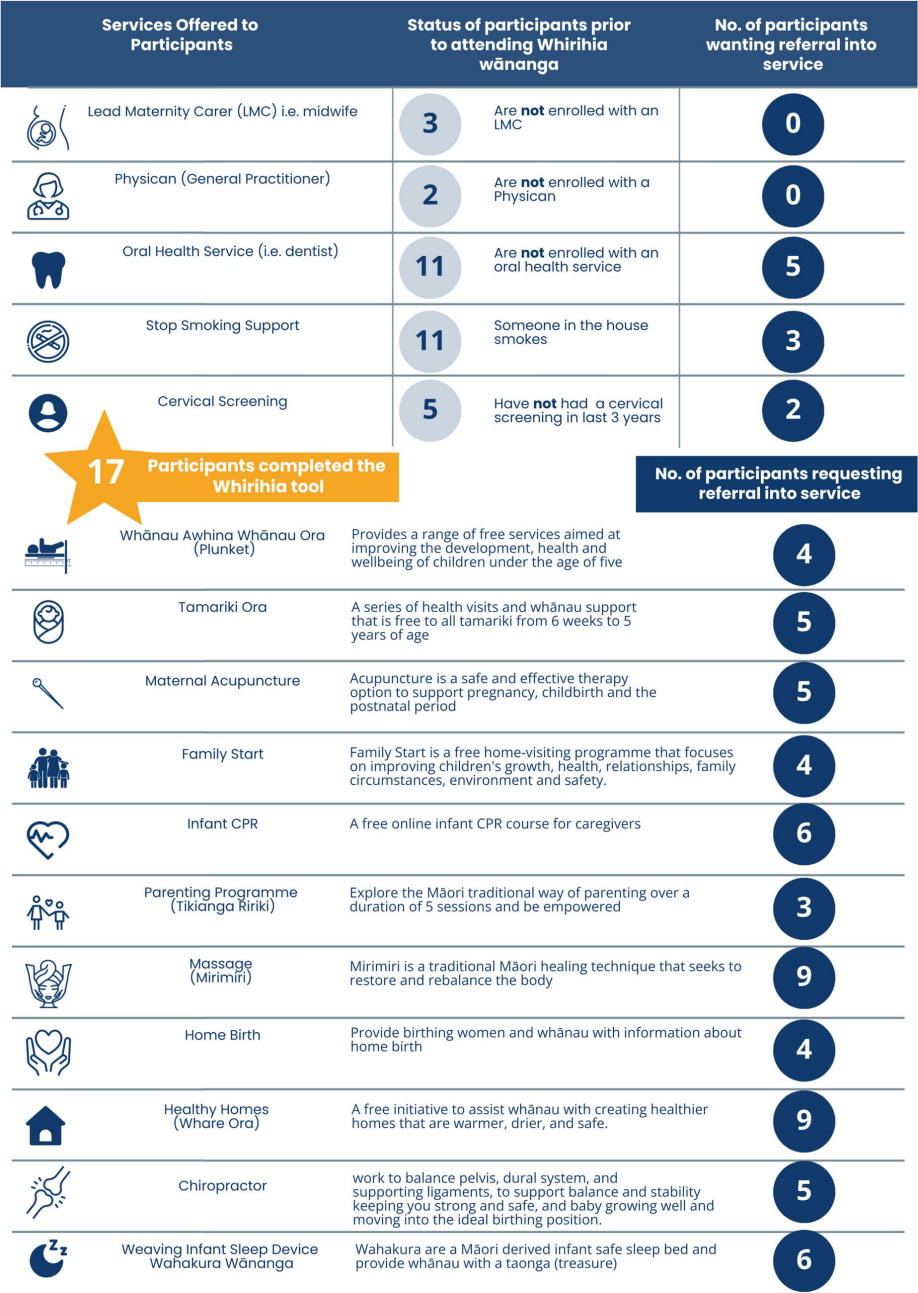
Phase one results

As part of a larger doctoral study, lead author NB conducted a three-phase small cohort study of māmā hapū (expectant mothers) recruited from the Kaupapa Māori childbirth education programme ‘Whirihia te Korowai Aroha (Whirihia)’ over 10 months. Figure 1 is a flow chart summary of the three phases. Participants had the option to exit the study at any phase. The first phase involved completing a co-designed holistic assessment tool [43]. The second phase was an electronic survey via Qualtrics. Data from the second phase informed the tailored interview questions for the third phase, which involved telephone interviews. This paper focuses on the third phase, aiming to understand how health services can better respond to Māori health needs and aspirations.

## Methods

This study is underpinned by Kaupapa Māori research, which is a methodological approach that centres on Māori worldviews, values, and cultural practices emphasising community participation, self-determination, and the prioritisation of Māori aspirations and needs. Using a mana wahine theoretical perspective that centres on Māori women’s experiences and knowledge, emphasising their authority and cultural identity within both historical and contemporary contexts, a thematic analysis was undertaken to amplify the lived experiences of seven Māori wāhine.

### Ethics

Approval from the University of Waikato Human Research Ethics Committee was granted 13 November 2019 HREC(Health)2019#40.

### Data Collection

As indicated in Fig. 1, the first data collection phase occurred in November 2019. Māmā hapū from the Whirihia programme were invited to participate in a University of Waikato research project that involved participants completing the Māori-led, and co-designed, Whirihia holistic assessment tool (Whirihia tool; see appendix one). The Whirihia tool featured a built-in referral pathway to wider health and social services.

A crucial component of Kaupapa Māori research is the process of connecting with participants (whakawhanaungatanga) by sharing genealogy and places of significance (i.e. where you were born or live). This practice took place face to face (kanohi ki te kanohi) to foster genuine connections and trust [44]. As Jones et al. [45] explain, ‘in order for a research project to achieve the best possible outcomes, those sharing and collecting information must be able to fully trust those who are ultimately responsible for the analysis, interpretation, reporting and dissemination of that data’ (pp. 68).

This process occurred whereby NB introduced herself. First with personal details such as genealogy (whakapapa) and locations attributed to lineage, and then with professional details such as a former health professional and current researcher. The details of what the project entailed for participants were then explained, specifically, the three phases of data collection.

The first phase involved the completion of the Whirihia tool at the end of the Whirihia wānanga. Of note, it was explained that participation was voluntary and those that did not wish to be part of the research were still able to complete the Whirihia tool and be referred into health and social services. This process of informed consent is a core characteristic of Kaupapa Māori research, whereby participants have a right to participate, withdraw, or decline participation in research [46]. Subsequently, participants were informed they would be contacted within 6 months to begin the second phase of data collection. Seventeen participants completed the Whirihia tool and consented to the research study.

The second phase involved the completion of an electronic survey via a Qualtrics link. A text message was sent to participants by NB inviting them to participate in phase two and three. Those that consented to participate were sent the survey link in June 2020, which was active for 4 weeks.

The survey asked participants to recall the services they had requested referral into from the Whirihia programme. We then asked participants to provide a brief outline of the positive and negative experiences of engaging with any health and social service, since the Whirihia class in November 2019. Responses from the survey were combined with open-ended questions to develop tailored talking points for the third data collection phase. Wāhine were reminded that participation was voluntary and at the completion of the survey, they would be contacted to undertake a phone interview. At the conclusion of the phone interview, a koha (token of appreciation) in the form of a voucher was given to participants.

The third data collection phase, which is the focus of this paper, involved telephone interviews conducted by NB. The original intent was to undertake kanohi ki to kanohi interviews with participants. This method of engagement is highly valued in Kaupapa Māori research practices, to support the continuity of building rapport and relationships with participants [46]. However, the impact of COVID-19 created a state of uncertainty and fear [47]. NB noted that as both a researcher and a mama (mother), she did not want to pose any health risk to participants; therefore, a phone interview was deemed most appropriate.

The interviews ranged in length from 25 to 55 min. Demographic data comprising of participants age, primp (first time pregnancy), and deprivation index number (the number allocated to geographical location; 1 refers to levels of low deprivation and 10 refers to levels of high deprivation) were collected (see Table 1). The ages of māmā varied with one māmā aged between 14 and 18 years, three aged between 19 and 25 years, one between 26 and 30 years, and two between 31 and 35 years. Three māmā had previous tamariki (children) whilst four wāhine identified as first-time mothers. Six of the participants resided in areas with a deprivation index of 9 and 10. The demographics of women were not a representation of the population. However, the demographics reflect a portion of the population who are often labelled ‘vulnerable’ due to the low deprivation characteristics.

**Table 1:** Demographics of participants: social deprivation area, age, and primp

**Demographic**		**Participant (*n* = 7)**
**Socioeconomic Deprivation Index**	10	2
	9	4
	3	1
**Age (years)**	14 to 18	1
	19 to 25	3
	26 to 30	1
	31 to 35	2
**Primp (first time pregnant)**	No	3
	Yes	4

The intentional design of tailored questions, based on individual survey responses, shaped the semi-structured interview questions. The tailored questions allowed participants to describe characteristics of their interactions with specific health and social services, that in turn shaped either a positive, negative, or in between experience. The semi-structured questions allowed for issues to be explored as they arose and enabled the conversation to progress naturally rather than being a rigid, structured process. The impacts of COVID-19 affected the logistics of our research team, whereby access to resources was limited. Interview notes were taken during, and at the conclusion of each interview; however, due to the limited resources available to NB, interviews could not be recorded. NB embedded a comprehensive process of reflexivity during the interviews, which involved constant confirmation with participants, to confirm accurate interpretation of data.

### Analysis

A thematic analysis process, using a mana wahine theoretical perspective, was undertaken by NB. The thematic analysis involved the categorising of patterns and generation of codes, eventuating into themes [48]. The thematic analysis identified commonalities and differences, within, and across, participants. The reading and re-reading of interview notes allowed for interpretations and meanings of the participants’ thoughts and experiences. The identification of repetitive themes reached saturation and five themes were identified. Pseudonyms were assigned to the seven participants.

## Results

### Phase One

**Fig. 2:**
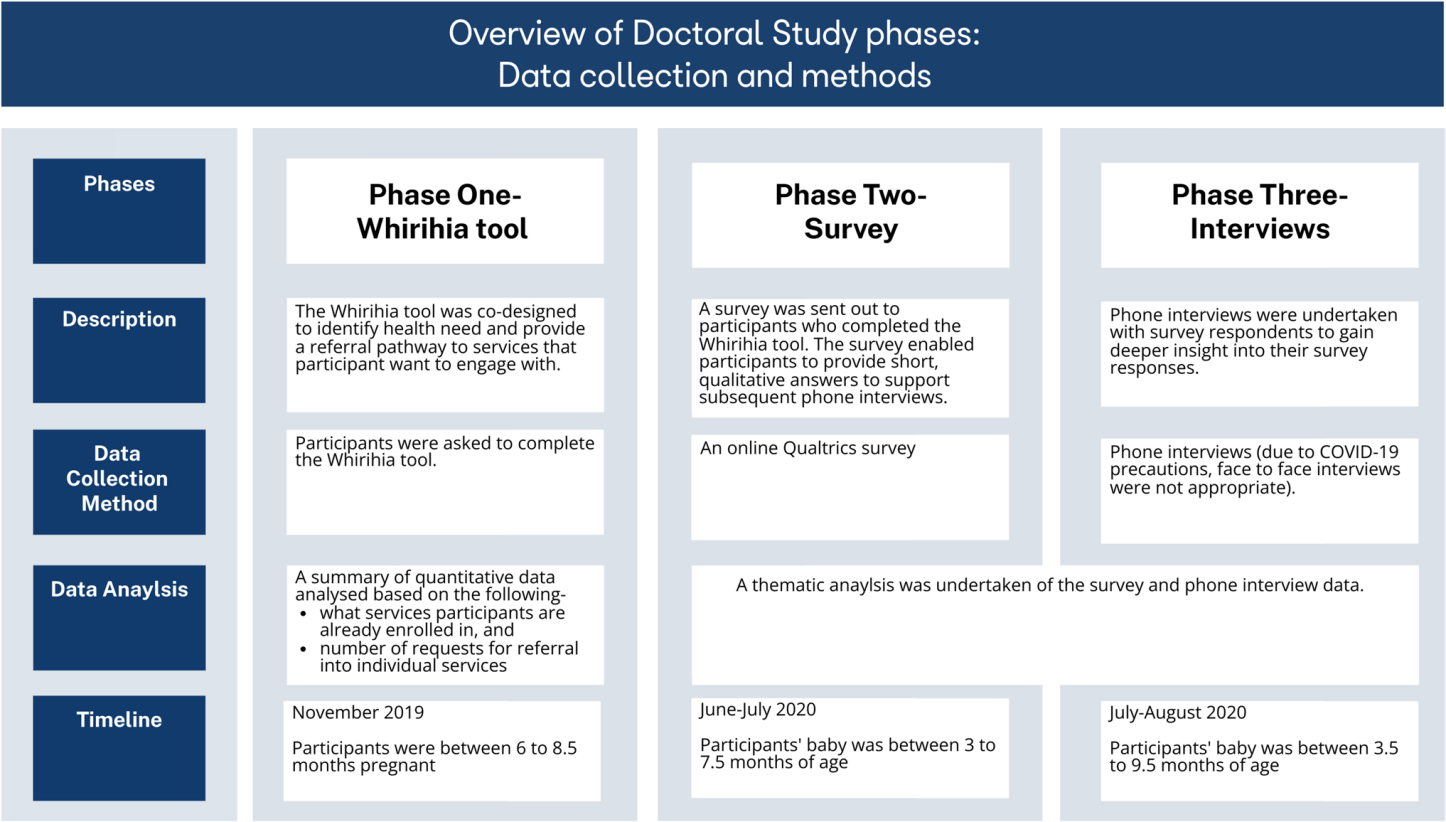
Overview of doctoral study phases

Twenty participants completed the Whirihia assessment tool. Figure 2 is a summary of the services offered to participants, an assessment of what services participants are already receiving, and number of participants requesting referral into each individual service.

### Phases Two and Three

On March 23, 2020, prior to commencing phase two, Aotearoa imposed a level 4 elimination strategy to address the introduction of COVID-19 in the country [47]. Data collected from surveys and interviews showed that the landscape of COVID-19 had dramatically changed health service delivery. As such, all participants noted a lack of engagement from multiple health and social services. The initial intent of the study was to determine the engagement experiences of each service that a participant was referred to via the Whirihia tool. As we could not assess this accurately, our research team decided to undertake a blanket approach of participant engagement experiences rather than service specific findings. Therefore, the following experiences are based on engagement with varying providers both included and excluded from the Whirihia tool.

**Fig. 3:**
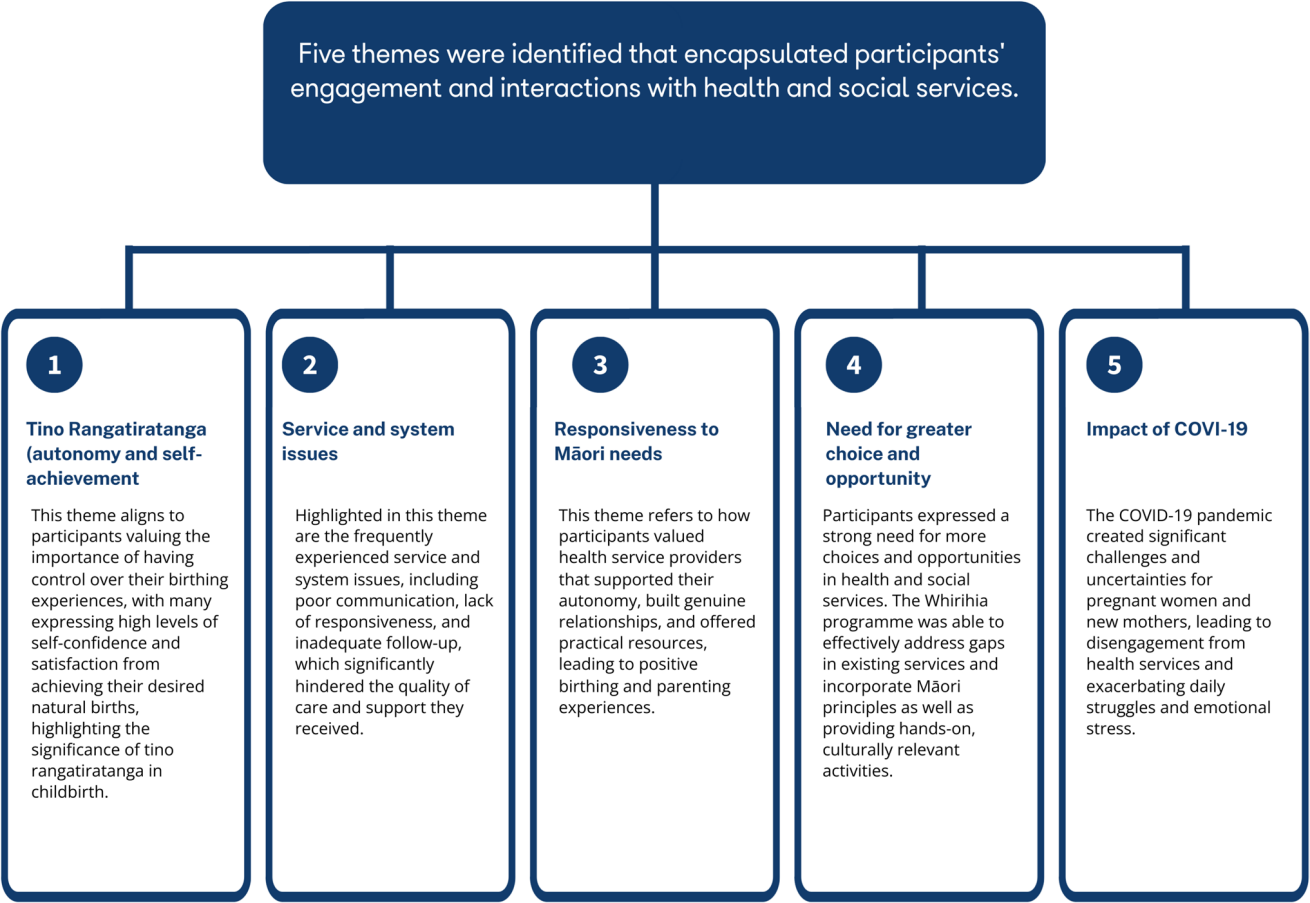
Flowchart of the five themes

Findings from the data analysis of the survey and phone interviews led to the establishment of five themes that encapsulated the experiences of participants’ engagement and interactions with health and social services. Figure 3 is a flowchart of the following five themes: (1) right to enact tino rangatiratanga (autonomy) and self-achievement; (2) responsiveness of services; (3) service and system issues; (4) need for greater choice and opportunity; and (5) impact of COVID-19.

#### Theme One: Tino Rangatiratanga (Autonomy) and Self-achievement

Each māmā expressed a desire to craft and enact their own birthing experience. As part of the reconnecting process and building greater rapport with participants, NB began each interview by inviting māmā to share their birth story. Each participant shared aspects of their birthing story, with many participants noting a positive labour and birthing experience. A high level of self-confidence in achieving their desired birth plan was noted by participants. Hinemoana explains one of the highlights of her birth story:

This was my first baby and it was a natural birth which is what I wanted. During labour I said I wanted an epidural but I didn’t end up doing it, yay!

Hinemoana reflected on her birth story with admiration for accomplishing her birth the way she planned. This admiration was shared by Anahera who was able to achieve the natural birth, without medical intervention, she had planned. Although Anahera required subsequent medical intervention after giving birth for second degree tearing, Anahera’s sense of self-accomplishment outweighed the subsequent medical intervention.

Aroha, Mere, and Amiria also shared similar experiences, each reporting positive, natural births, with Aroha and Amiria having planned home births. These experiences, coupled with Hinemoana and Anahera’s stories, demonstrate that the ability to enact tino rangatiratanga over birthing choice is of great importance.

#### Theme Two: Responsiveness to Māori Needs

This theme explores the features of health service provision that contributed to participants having a positive experience during their birthing or parenting journey. Some health service providers created an environment for māmā to practice tino rangatiratanga. The Whirihia programme for instance enabled expectant māmā to choose if, and what services, they wanted to engage with. This approach was well received by participants. Anahera for instance was able to access a range of health providers that she was unaware of prior to attending the Whirihia class, one being maternal acupuncture, which aligned well with her aspirations for birth. Hoping to avoid complications during birth, Anahera found acupuncture a natural option to support this outcome. Anahera noted that she ‘loved it’ although the cost to access this support was steep. Anahera exclaims, ‘I had a good birth outcome and pretty sure [acupuncture] made labour easy’.

After giving birth to her pēpi, Aroha described her stay with a local birthing unit as ‘amazing’. Aroha expanded on why she described her experience this way and gave specific examples to illustrate how this birthing unit shaped her experience.

The midwife was amazing. The attentiveness of staff, nurse always checking on us. Not intruding, just there to help. They genuinely wanted to get to know us, not tick box. Food was great, actually amazing. [They] left us to ourself, could do our own thing. Gave us space to bond.

The quote by Aroha demonstrates that the birthing unit provided a supportive role for both Aroha and her whānau rather than using an authoritative approach. This approach created an environment where Aroha was able to make decisions without feeling pressure from outside influences. The feeling of being genuinely cared for by services was highly valued by Aroha and other participants as outlined in the subsequent examples.

Well Child Tamariki Ora (WCTO) provides universal early childhood health and development services to all tamariki (children) under 5 years and their whānau [42]. Hinemoana shared her positive experiences engaging with a Māori WCTO provider, describing the service as more engaging than a previous non-Māori WCTO provider her pēpi was enrolled in.

Instant reply to my text messages. When she [WCTO staff] came to my house she answered my questions. She gave me a book and bath mat. I didn’t get any resources from [previous WCTO provider] even though I was told I should have by [current WCTO provider].

Practical resources were valued by some participants. Mere enjoyed her interaction with a WCTO provider as they were able to provide her with baby clothes and a car seat. This was particularly useful given both her and her partner had no income at the time. Maanaki found one service ‘really cool’ as they supported her and her children with heaters, beds, and bedding. This service also supported Maanaki to liaise with Housing New Zealand (social housing agency) and advocate for cost-effective heating sources.

Alongside practical resources, participants highly valued the genuine connection and relationship provided by services. As Hinemoana exclaims why she valued her interaction with her WCTO provider. ‘Their willingness to actually help. Be there to answer questions’—Hinemoana. Maanaki valued the ongoing relationship she had with her Māori midwife who had birthed her previous children, and the ability to ‘just click’ with her.

For Aroha, one service provided a space of support for her pēpi, instilling reassurance and confidence that her pēpi was being cared for.

Baby is 7 months and is in daycare and daycare is fabuolous. Would not take my baby anywhere else, that’s why we have to stay in [local]. The daycare is a bit over the top actually ‘Diligent’ ‘[it’s a] necessity to have a good place’.

#### Theme Three: Service and System Issues

Though māmā identified positive aspects of their interactions engaging in services, several service and system issues reoccurred in each of the shared experiences, resulting in a lack of responsiveness from services.

Mere noted she had a great natural birth but then a bad experience when her pēpi had to be taken to the Newborn Intensive Care Unit (NICU).

I had a great birth, natural. But then we had to go to the hospital soon after baby was born to NICU. Heaps of blood tests. Procedure wasn’t sterile and baby got an infection. The infection went into his heart and lungs, they put an infection in him. The doctors were incompetent. The communication amongst each other was terrible. Baby couldn’t have general anaesthetic but the doctor and nurses didn’t tell the anaesthetist, and was going to give it to him. My mum had to prompt the doctor and he went to check.

Mere further explains that the bad communication amongst the staff and with her whānau exacerbated the negative experience. A lack of clear and informative communication was present in other participants’ experiences. Hinemoana described her interactions with a non-Māori WCTO nurse:

There were no text messages or calls back. She [WCTO nurse] just came around and couldn’t answer any of my questions, it was horrible. I couldn’t understand the nurse. You felt like she was just there to do what she needed and didn’t care about what I needed.

Maia echoed similar sentiments, sharing that her non-Māori WCTO nurse was not able to provide accurate and relevant content in a timely manner.

They [WCTO nurse] told me things I already knew. It felt really structured they just keep saying, refer to your GP, so I gave up as I was not getting answers for what I wanted. It felt like a tick box, did this, do that. I said to the nurse, maybe you should have these as frequently asked questions, I know other mums want to know this.

These examples shared by Hinemoana and Maia demonstrated that a lack of empathy and care expressed by the health service providers resulted in unmet need. As a consequence, Maia noted that she ‘gave up’ as she was not getting the answers she required. Anahera shared a similar experience with a WTCO nurse that resulted in her feeling an inability to be honest with her WTCO nurse for fear of repercussions.

I told her that I sleep with baby, got a huge lecture, now I just lie to her. It got to the point where I was like, you can pass this info onto someone else.

Though the examples noted above highlight a lack of responsiveness of service delivery, some māmā received no engagement from any health or social services after the birth of their pēpi. When questioning Aroha as to whether she had received contact from services, her response was:

Kao [no]! No services have engaged with me, heard nothing. I feel that because I didn’t have a normal ‘mainstream’ birth I was lost in the system, and because of covid. I was a bit disappointed though as I talked with a lot of the services at the [Whirihia] wānanga [workshop] and no one has contacted me. My midwife was awesome, and she made referrals to [four services] but still no follow up.

Aroha’s experience demonstrates a severe flaw in service provider referral processes. There were two key points where access barriers could have been addressed, at the Whirihia programme, and again when her midwife referred her to four distinct services. Yet, her pēpi is now 7 months old and she has yet to receive engagement from any health or social service.

Mere reaffirmed this flaw in service provider referrals explaining that since her pēpi was born she has only received one phone call from a WCTO provider, and no other services. Similarly, Maia noted that she did not receive contact from WCTO services during lockdown and had to initiate engagement with the service.

Though Aroha and Amiria have other tamariki (children), they each highlighted that previous parenting experience does not mean they do not require support, as each pēpi has their own unique needs. As Aroha notes:

She is my fourth baby, lucky I had experience. She is in good health, feeds good, didn’t really need much but even if it’s your 10 th baby, all babies are different and it takes a village not just an experienced mum.

#### Theme Four: Need for Greater Choice and Opportunity

Highlighted in many of the participant interviews was a need to have greater choice of health and social services, and opportunities to engage in said services. Hinemoana explains that she attended the Whirihia programme because there were no similar programmes offered where she resides. Hinemoana described her reaction to the Whirihia programme:

I loved it. I loved the hands-on parts of the workshop, making the placenta bowls, the weaving…. My doctor just gave me pamphlets and said read this.

Amiria also found value in the Whirihia wānanga and because it was offered in her locality she attended. The factors of the wānanga that Amiria valued included:

Māori tikanga (principles and protocols), getting to know other Māori māmā, [and ability to] grab the bits I needed to know.

The preceding narratives demonstrate that the Whirihia wānanga filled a gap for these wahine that other services were not providing. Manaaki shared similar sentiment and goes further with a suggestion to expand the wānanga to fill a greater unmet health need:

My friend told me about it [Whirihia wānanga]. Was an amazing two days. Would love to attend a focus wānanga group and meet up with everybody again.

Some māmā were unaware of the different health and social services available in their locality. Hinemoana for instance changed into another WCTO provider after her cousin informed her, she was able to do so. The new service was a Māori WCTO that Hinemoana found greater connection and responsiveness with.

Mere was also with a Māori WCTO provider and although she had to travel to engage with the service, she reported that she was happy to do so. Anahera reported that because of the Whirihia wānanga, she was able to connect to maternal acupuncture services and intends on enrolling in infant CPR after becoming aware of the service.

#### Theme Five: Impact of COVID-19

Participants were asked to describe their experience with health and social services during and after pregnancy. Aroha explained that she had received no engagement with services. Aroha identified that the COVID-19 pandemic was one potential factor for non-engagement.

Due to covid-19 lockdown, all non-essential services ceased to operate so we weren’t sure when and how they’d start back up.

The lack of communication from services created uncertainty for Aroha, and other participants. Yet, despite having no engagement from services, Aroha appreciated and understood that COVID-19 impacted health services and that these services faced significant challenges.

Amiria shared the impacts the COVID-19 pandemic has had on her and her whānau.

You just exist! Everyone should have equal. On benefits people get more. I was on maternity pay and it was small but I need to work to top it up. I can’t afford day care but I need to work. It’s a problem to get ourselves stuff, we are not in poverty, don’t have domestic violence. I was working through Covid but not entitled to resources.

Amiria revealed a range of emotions she has experienced as a result of the COVID-19 pandemic. The daily struggles and realities of parenting coupled with the impacts of the pandemic have created an atmosphere of fear and uncertainty. Amiria disclosed numerous issues she is struggling with including balancing work and parenting obligations.

## Discussion

The experiences of Māori mothers shared in this study highlight the critical importance of culturally appropriate healthcare for improving Māori perinatal health. Participants highlighted actions taken by health services that enhanced their maternal and postnatal journey, including, providing holistic care that respects the autonomy and choices of women, and establishing rapport to build strong, trust-based relationships. Furthermore, participants valued genuine connections with providers who displayed compassion, warmth, honesty, and respect. These findings are consistent with Māori women’s broader experiences of health with Masters-Awatere and Graham [26] revealing that ‘…the combination of listening work, self-autonomy and genuine care for their wellbeing as Māori left participants feeling valued’ (p. 473). Similarly, Walker et al.’s [49] study highlighted that displays of compassion, warmth, honesty, and respect were highly valued by Māori patients. These characteristics have been echoed in by international scholars, such as Seear et al. [50] who found that Aboriginal women in the Kimberley region of Western Australia, had a positive antenatal care experience when the antenatal care provider created a trusting relationship and built rapport with women.

Consistent with findings from previous studies on Māori women, as well as international scholarship on Indigenous Peoples experiences of maternity care [51–53], our findings revealed participants had greater incidences of negative experiences when engaging with service providers. Negative experiences were linked to poor quality service delivery, such as dismissive attitudes and inappropriate information, leading to distrust and adverse outcomes. Graham and Masters-Awatere’s [54] systematic review found that the ‘absence of relational connection contributed to ongoing negative narratives between patients and health workers’ (p. 197). Jansen et al.’s [55] findings had similar results with Māori reporting being talked down to or treated with disrespect by staff. These findings are a reoccurring theme in several studies on Indigenous birthing women of Aotearoa, Australia, Canada, and the USA [50–52, 56].

These four nations share similarities; due to colonial incursion, still there are unique factors that continue to impact Indigenous birthing women. For instance, in the 1970 s, extremely large numbers of Native American women suffered involuntary sterilisation [57], an experience First Nations women of Canada also suffered [58]. First Nations women in Canada who live on rural and remote reserves must also endure relocation, having to leave their communities between 36- and 38-week gestational age to attend urban centres to labour and birth, creating emotional, physical, and financial stress [59]. The vast rural and remoteness of Aboriginal women in Australia means there is a severe lack of quality and responsive care for birthing women [50]. These examples have created deep seeded mistrust with Western health services and continue to play a significant role in poor maternal outcomes.

To counter past systematic failures, Māori scholars argue that exemplar pathways of care for pregnant Māori women and their whānau would be engagement with Māori services anchored in a Māori world view (te ao Māori) [2, 37, 60, 61]. These services use Kaupapa Māori principles as the foundations of service delivery and practice. For instance, Tapuhi Ahi Kā, a newly established service, combines 40 community-based Māori health partners across Aotearoa, with a midwifery-led, wrap-around service for hapū māmā and their whānau, to enable continuity of care throughout pregnancy, birth, and a child’s early years [62]. Initiatives, such as the Whirihia programme [43] or Hapū Wānanga [63], are examples of blending Māori philosophies and understandings of health with Western medical practices, resulting in higher participation rates and community acceptance and endorsement. These approaches to maternal health care resonate with other Indigenous Peoples with a study in Canada demonstrating that a community-led educational programming for Indigenous populations was linked to a positive change in birth outcomes, access to prenatal and postnatal care, prenatal street drug use, breastfeeding, dental health, infant nutrition, and child development [64].

Though studies have evidenced the acceptability of Indigenous-led services, similar to the Indigenous Peoples of Australia, Canada, and the USA, Māori consumers lack choice when it comes to participating in programmes or services. Participants from this study identified a lack of awareness regarding what services are available as well as a lack of culturally appropriate programmes. The low number of Māori health professionals compared to non-Māori means whānau have difficulty engaging with Māori practitioners. In 2019, there was a total of 3226 practicing midwives, of which 317 (9.83%) identified as Māori [65]. The demand for midwives is high particularly for Māori midwives, with many experiencing exhaustion from being under-resourced and subjected to bullying behaviour by those who obstruct, side-line, and drive out Māori colleagues that challenge the status quo or speak up for change [65]. Though there are initiatives to grow the Māori midwifery and greater Māori health workforce [66], women often have no alternative but to enlist a non-Māori midwife and engage in non-Māori services.

The lack of Indigenous maternity staff is an issue shared with other Indigenous Peoples. For instance, Marriott et al. [67] identified access to Aboriginal health professionals and services in rural Australia as a key barrier for birthing women to receive culturally appropriate care. Further studies have recognised that integrating traditional practices and beliefs into health services fosters trust and respect between patients and providers, leading to higher satisfaction, increased engagement, and better health outcomes [68, 69]. This approach is particularly relevant for non-Indigenous services, whereby implementation of these practices can significantly enhance engagement and outcomes [70]. Acknowledging the need for cultural safety in maternity care, the Midwifery Council of New Zealand recently released the recertification programme for the next 3 years, and for the first time, it includes compulsory cultural safety education. This means midwives will undertake ongoing education in te ao Māori, Te Tiriti o Waitangi, and culturally safe practices [71].

To ensure services and providers are meeting their obligations to achieve maternal and infant health equity, effective monitoring and evaluation is needed. Indigenous evaluation frameworks are essential for ensuring health services are culturally relevant and respectful of Indigenous values [72]. Kaupapa Māori Evaluation (KME) is a process that can be implemented into health intervention programmes to ensure a culturally appropriate assessment is undertaken. Carlson et al. [73] argue that KME can meet the ‘aspirations of co-ownership, mutually beneficial outcomes and shared power’ (p. 1). KME also considers evaluation processes that recognise Māori values, self-determination, and aspirations. Using KME with a mana wahine approach can challenge dominant hegemonies but most importantly can validate mātauranga Māori (Māori knowledge).

The need for evaluations contributes to the wider issue of a lack of Māori health service options, such as a lack of Māori midwives or Kaupapa Māori parenting programmes [4]. Māori-led interventions are more inclined to have less resourcing support than Western designed interventions [41]. The lack of support afforded to Māori-led interventions perpetuates the use of universal programmes with a ‘one-size fits all approach’ that does not work for Māori [74].

The Waitangi Tribunal [4] report presents evidence that despite the obligations of Te Tiriti o Waitangi, one reason for the insufficient number of Māori health services is a result of ongoing failures in investment processes determined by Government agencies. To invest in Māori health, the Health and Disability System Review [75] and Came et al. [8] highlight the need to identify what, and where, new investment and disinvestment should occur. To understand which programmes, require new or disinvestment, programmes require monitoring and evaluation to ensure they are meeting the needs of end-users.

As highlighted in this study, the COVID-19 pandemic has exacerbated existing inequities in Indigenous pregnancy and birthing experiences by disrupting health services and reducing access to prenatal and postnatal care. This has negatively affected Māori mothers and infants, reflecting global trends where Indigenous populations faced significant barriers to timely and culturally sensitive healthcare [42, 76, 77]. Pregnant women in Indigenous communities faced heightened risks due to disrupted healthcare services, increased stress, and limited access to culturally appropriate care, leading to higher rates of anxiety and poorer health outcomes [42, 78, 79].

Participants in this study disclosed challenges faced that have negatively impacted their financial, emotional, and social wellbeing. These findings align to Hannah et al.’s [80] report explaining that COVID-19 has impacted Māori communities, contributing to financial hardship, issues of housing, negative impacts on wellbeing, and increased transmission of vaccination misinformation and disinformation. These issues, including non-engagement from health services, are not new challenges created by the COVID-19 pandemic. Pihama and Lipsham [81] explain that these challenges have been struggles Māori have faced for generations and further stresses the need for Māori be prioritised to ensure equity gaps are not further widened.

## Limitations

A limitation of this study is the low number of participants to provide an accurate representation of Māori wāhine. Whilst there is growing scholarship on Māori wāhine experiences of health care, there is limited knowledge of how these experiences influence health policy and practice. The points raised in this paper pertaining to greater choice of Māori interventions and need for appropriate evaluation to provide investment, or disinvestment, are health system issues. As per the obligations of Te Tiriti o Waitangi, that reaffirmed Māori tino rangatiratanga and promised equity (ōritetanga) [7], Māori wāhine experiences and voices must be central to health system decisions. Therefore, this paper makes a necessary and timely contribution to enhance and improve health services, to benefit Māori māmā and further suggests in depth exploration in this area.

## Conclusion

This paper has sought to enhance understanding of Kaupapa Māori within the maternal space, specifically its significance in shaping the design of health service delivery and application to research. To understand, address, and ultimately eliminate ethnic health disparities in Aotearoa, Māori experiences must be prioritised.

The voices of Māori wāhine have been silenced for too long and have resulted in the silencing of their theories and world views. Using a mana wahine approach, this study privileged the voices of these wāhine and their experiences navigating a complex, and often, irresponsive health system. Tino rangatiratanga was at the forefront of positive participant experiences. When services enabled participants to enact autonomy and self-determination participants valued their engagement with that service. The guarantee of tino rangatiratanga provides for Māori self-determination and mana motuhake in the design, delivery, and monitoring of health and disability services.

To support Māori māmā to have positive experiences with health and social services, this paper identified key service provider delivery barriers and health system failures that require strengthening and are so mandated by Te Tiriti o Waitangi. These include the need for services to provide responsive, appropriate, and timely information. Also, services need to create genuine connections with māmā, to instil a sense of trust for māmā to be supported to approach services with their concerns and subsequently receive the necessary care to ensure optimal health.

Services that connected with participants and demonstrated a genuine interest for the emotional and physical wellbeing of māmā and pēpi resulted in a positive experience. If services showed empathy and compassion for māmā and operated in a passive role of support rather than a position of authority and power, services would be more responsive.

## Supplementary Information

Below is the link to the electronic supplementary material.

Supplementary file1 (PDF 29610 KB)

## Glossary

Māori: Indigenous Peoples of Aotearoa/New Zealand
Aotearoa: Māori name for New Zealand
Pākehā: Non-Māori, predominately European descent
māmā: Mothers
mana wahine: Authority inherent in Māori women
tino rangatiratanga*: Autonomy
Tohunga: Māori healer
marae: Ancestral home
pēpi: Baby
tikanga: Cultural protocols, principles, and beliefs
wāhine: Women
tapu: Sacredness
māmā: Mothers
Whirihia te Korowai Aroha (Whirihia): Kaupapa Māori childbirth education class
māmā hapū: Expectant mothers
whanaungatanga: (A process of introducing and connecting with others, often based on genealogical connections)
kanohi ki te kanohi: Face to face
whakapapa: Genealogy
koha: Token of appreciation
mama: Mother
tamariki: Children
wānanga: Workshop
mātauranga Māori: Māori knowledge
ōritetanga: Equity
