# Supplementary material for: Experiences of New Zealand Māori Mothers’ Engagement with Health and Social Services Post-COVID-19 2020 Lockdown
Source: J Racial Ethn Health Disparities. 2025 Apr 22;13(3):2304–17. doi: 10.1007/s40615-025-02419-4 (PMC13157368; doi:10.1007/s40615-025-02419-4)

Whirihia Holistic Assessment tool

plunket
whānau āwhina

Personal Details

First and Surname: ______________________

Preferred name (if applicable): ______________________

NHI number (if known) ______________________

Date of Birth: ______________________

Which ethnic group do you belong to? (tick as many that apply to you)

- ☐ New Zealand European
- ☐ Māori
- ☐ Samoan
- ☐ Cook Island Māori
- ☐ Tongan
- ☐ Niuean
- ☐ Chinese
- ☐ Indian
- ☐ Other- Please State: ______________________

If Maori what is you iwi/hapū/marae? (write as many that apply to you)

Iwi: ______________________

Hapū: ______________________

Marae: ______________________

Contact phone number: ______________________

Address

Number and Street: ______________________

Suburb: ______________________

Do you have a community services card?

☐ Yes ☐ No ☐ Unsure ☐ Prefer not to answer

Baby due date:

Do you currently have any other tamariki enrolled in Plunket?

☐ Yes ☐ No ☐ Unsure

Child/s name ______________________

Date of Birth ______________________

Research Consent

Content on this page
has been removed for
publciation purposes

THE UNIVERSITY OF
WAIKATO
Te Whare Wānanga o Waikato

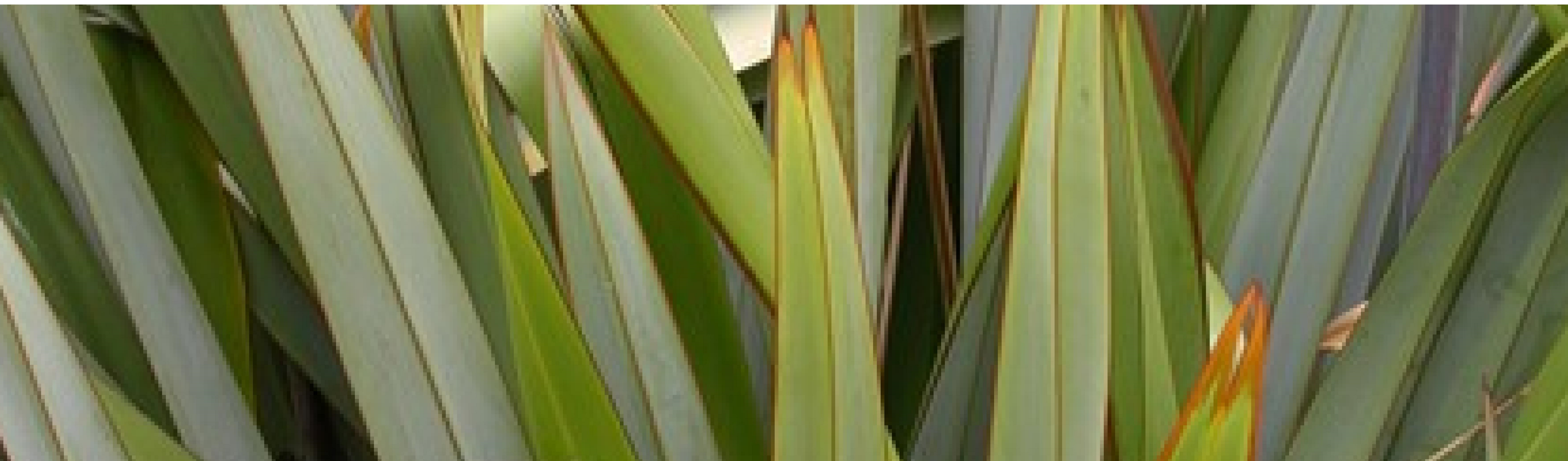

Current health status and referral

If you tick 'yes' you would like a referral into any of the following services we will send the details you have provided direct to the service.

Question	Answer	Follow-up question	Next
1. Do you have an LMC?	☐Yes but thinking of changing<br>☐No<br>☐Unsure	→ 1b Would you like to be referred to a LMC?<br>☐ Yes<br>☐ No	→ Go to question 2
	☐ Yes<br>☐ Prefer not to answer		→ Go to question 2
2. Are you currently enrolled in a GP service?	☐ Yes but thinking of changing<br>☐ No<br>☐ Unsure	→ 2b Would you like to be referred to a GP clinic in your area?<br>☐ Yes<br>☐ No	→ Go to question 3
	☐ Yes<br>☐ Prefer not to answer		→ Go to question 3
3. Are you currently enrolled with a dentist?	☐ Yes but thinking of changing<br>☐ No<br>☐ Unsure	→ 3b Would you like to be referred to the oral health service?<br>☐ Yes<br>☐ No	→ Go to question 4
	☐ Yes<br>☐ Prefer not to answer		→ Go to question 4
4. Do you or someone you live with smoke?	☐ Yes	→ 4b Would you like to be referred to the quit smoking service?<br>☐ Yes<br>☐ No	→ Go to question 5
	☐ No<br>☐ Prefer not to answer		→ Go to question 5
5. Have you had a 'smear' test in test last 3 years?	☐ No<br>☐ Unsure	→ 5b Would you like to be referred to the cervical screening service?<br>☐ Yes<br>☐ No	→ Go to next page
	☐ Yes<br>☐ Prefer not to answer		→ Go to next page

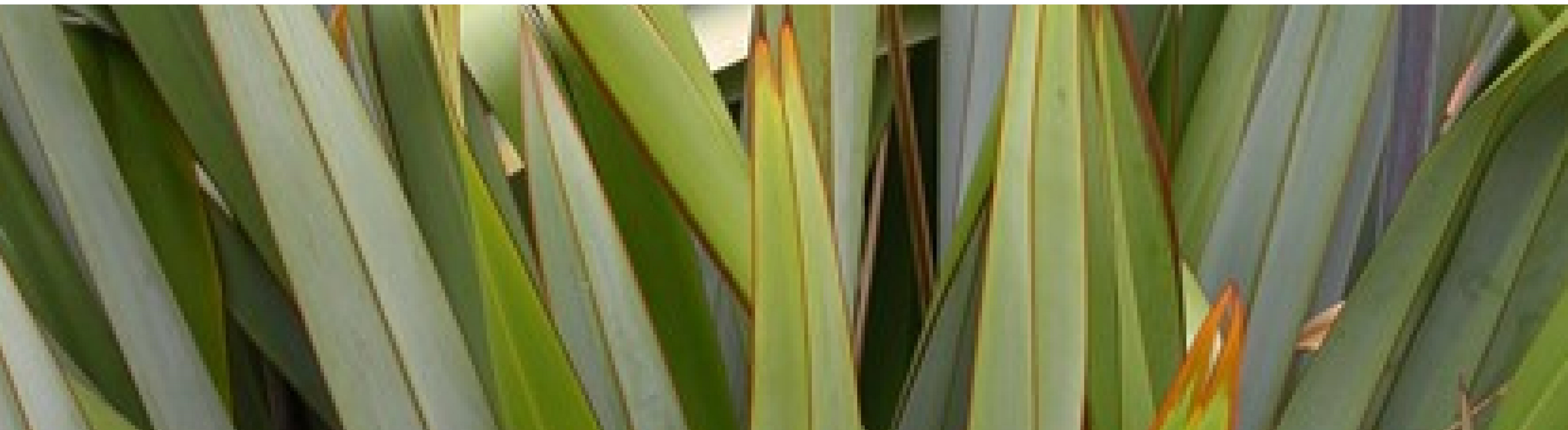

Tick the following boxes if you would like a referral into any of the following services

Whare Ora Programme
☐ Yes

Infant CPR Programme
☐ Yes

Home Birth service
☐ Yes

Whānau Awhina Whānau Ora (Plunket) Programme
☐ Yes

Tamariki Ora Programme
☐ Yes

Wahakura Wānanga
☐ Yes

Maternal Acupuncture Service
(There is a cost associated with this service)
☐ Yes

Phillips Family Chiropractor
(There is a cost associated with this service)
☐ Yes

Mirimiri service
(There is a cost associated with this service)
☐ Yes

Family Start or
Kirikiriroa Family Service Programme
☐ Yes

Tikanga Ririki Programme
☐ Yes

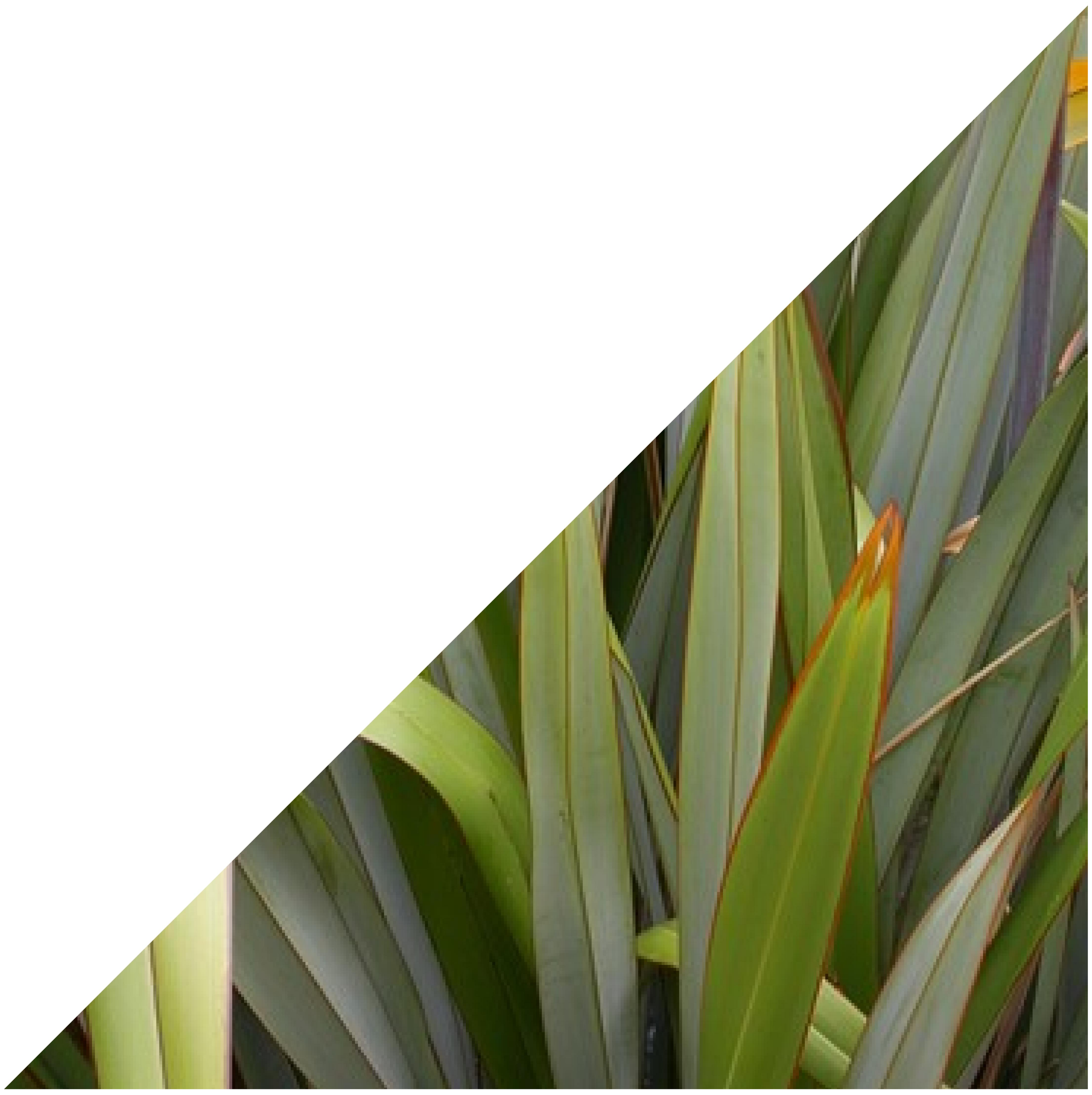

Supplement: Supplementary file 1 — Supplementary file1 (PDF 29610 KB) [file 40615_2025_2419_MOESM1_ESM.pdf]
